# Heavy Metal Ion Stress on *Halobacterium salinarum* R1 Planktonic Cells and Biofilms

**DOI:** 10.3389/fmicb.2018.03157

**Published:** 2018-12-18

**Authors:** Sabrina Völkel, Sabrina Fröls, Felicitas Pfeifer

**Affiliations:** Microbiology and Archaea, Department of Biology, Technische Universität Darmstadt, Darmstadt, Germany

**Keywords:** PMA-qPCR, metal resistance, cell vitality, haloarchaeal biofilms, gene expression

## Abstract

*Halobacterium salinarum* R1 is an extremely halophilic archaeon, able to attach to the surface and to form characteristic biofilm structures under physiological conditions. However, the effect of environmental stress factors like heavy metals on biofilms was still unknown. Here, we report on the first insights into *H. salinarum* biofilm formation when exposed to copper, nickel and zinc and describe the effects of metal ions on the architecture of mature biofilms. We also studied the effects on gene expression in planktonic cells. Investigation of planktonic growth and cell adhesion in the presence of sub-lethal metal concentrations yielded an up to 60% reduced adhesion in case of copper and a significantly enhanced adhesion in case of zinc, whereas nickel treatment had no effect on adhesion. A PMA-qPCR assay was developed to quantify live/dead cells in planktonic cultures and mature biofilms, enabling the investigation of cell vitality after metal exposure. An increased resistance was observed in biofilms with up to 80% in case of copper- and up to 50% in case of zinc exposure compared to planktonic cells. However, nickel-treated biofilms showed no significant increase of cell survival. Microscopic investigation of the architecture of mature biofilms exposed to lethal metal concentrations demonstrated an increased detachment and the formation of large microcolonies after copper treatment, whereas the number of adherent cells increased strongly in nickel-exposed biofilms. In contrast, zinc exposed-biofilms showed no differences compared to the control. Analysis of the expression of genes encoding putative metal transporters by qRT-PCR revealed specific changes upon treatment of the cells with heavy metals. Our results demonstrate diverse effects of heavy metal ions on *H. salinarum* and imply a metal-specific protective response of cells in biofilms.

## Introduction

Microbial biofilms are the predominant lifestyle of microorganisms in natural habitats. The formation of biofilms is initiated by the adhesion of cells to a surface, followed by proliferation, formation of microcolonies and the production of extracellular polymeric substances (EPS). The EPS matrix surrounding the cells consists mainly of proteins, polysaccharides, lipids, and nucleic acids (Davey and O’Toole, 2000). This mode of life offers several advantages including increased nutrient supply, intercellular connections, as well as an enhanced resistance to antimicrobial agents (Flemming and Wingender, 2010). Biofilm-mediated resistance to heavy metal ions has been studied in detail for bacteria and some archaeal species (Koechler et al., 2015; van Wolferen et al., 2018). The influence of heavy metal ions varies within a millimolar range between growth stimulating and toxic effects on cells (Geesey and Jang, 1989), and therefore the metal concentration in the cellular environment is crucial. Compared to the planktonic lifestyle, cells in biofilms benefit from several advantages. The negatively charged EPS matrix binds positively charged substances such as metal ions and serves as a diffusion barrier restricting or at least slowing down the penetration of ions into the biofilm (Hullebusch et al., 2003; Harrison et al., 2007). Adaptations include alterations of the amount and composition of the EPS matrix (Priester et al., 2006; Marchal et al., 2011) or a slowdown of growth due to a reduced metabolic activity of the cells in biofilms (Costerton et al., 1999; Lewis, 2001). Persister cells, i.e., a small population of cells with even slower metabolic activity, are mainly produced in the stationary phase and also in biofilms (Harrison et al., 2005). These cells survive in the presence of toxic agents as shown for *Xylella fastidiosa* biofilms exposed to copper (Muranaka et al., 2012).

Work on the effect of heavy metal ions on microbial biofilms is almost limited to bacterial biofilms, whereas archaeal biofilms are much less studied. Metal-induced biofilm formation was observed in the hyperthermophilic euryarchaeon *Archaeoglobus fulgidus* exposed to chromium, copper and nickel (Lapaglia and Hartzell, 1997). The effects of heavy metals on halophilic archaea are of particular interest. Their natural habitats, like estuaries or salt crystallizer ponds, contain up to 5 M sodium chloride, and are often contaminated by heavy metals due to anthropogenic activities like urbanization, industrialization or mining (Srivastava and Kowshik, 2013). Previous studies focusing on the occurrence of heavy metals in these habitats describe iron concentrations in the molar range, whereas nickel-, zinc-, cobalt-, copper-, manganese-, lead- and cadmium concentrations are in the micro- to millimolar range (Kumar et al., 2010; Pereira et al., 2013). Studies on haloarchaea concerning the effects of metals are limited to the determination of the minimal inhibitory concentrations of *Halobacterium*, *Haloferax*, *Halorubrum*, and *Haloarcula* grown in liquid cultures (Nieto et al., 1987), whereas biofilm formation and biofilm-mediated resistance were neglected. To investigate metal resistance in haloarchaeal biofilms, a suitable method for live/dead quantification is required.

Traditional culture-based approaches to determine live/dead cells are limited to quantify the amount of culturable cells, whereas cells in the “viable but non-culturable” (VBNC) cell state were not taken into account (Oliver, 2005). Nucleic acid-based techniques, like polymerase chain reaction (PCR) have been developed to identify and quantify microorganisms (Boutaga et al., 2003; Suzuki et al., 2004). However, DNA persists for long periods of time after cell death, leading to an overestimated number of viable cells after antimicrobial treatment. Methods based on the membrane integrity of cells, including live/dead staining and fluorescence microscopy, are common approaches to differentiate live and dead cells. However, it is difficult to detect and differentiate live and dead cells in dense biofilm structures by microscopy. An approach based on cell treatment with the membrane-impermeable dye propidium monoazide (PMA) in combination with qPCR is a promising method for live/dead quantification of cells in biofilms. PMA selectively permeates the membrane of dead cells and intercalates into the DNA helix. After photoactivation, PMA covalently binds to DNA and inhibits its amplification, enabling live/dead quantification in a subsequent qPCR assay (Nocker et al., 2006). This method was successfully applied in studies concerning live/dead quantification of bacterial species (Àlvarez et al., 2013; Sánchez et al., 2014), but is not applicable at high salt concentrations required for haloarchaea (Barth et al., 2012).

The aim of the present study was to investigate the effects of the heavy metal ions copper, nickel and zinc on *Halobacterium salinarum* R1 with regard to surface adhesion and survival of cells in mature biofilms. Metal-specific responses of the cell adhesion were observed when the cells were treated with one of these ions. To determine the cell survival in biofilms, the PMA-qPCR approach was adjusted to the haloarchaeon *H. salinarum.* Different effects on adhesion were observed with *H. salinarum* when exposed to low concentrations of metal ions. The exposure of cells to toxic metal concentrations resulted in differences in cell survival in biofilms compared to planktonic cells. In addition, the effect of these metal ions on the structure of biofilms was analyzed by confocal laser scanning microscopy, and the expression of genes encoding several transport systems was investigated by qRT-PCR.

## Materials and Methods

### Cultivation Conditions

*Halobacterium salinarum* R1 (ATCC 2934) was grown at 37°C in complex medium (4.3 M NaCl, 81 mM MgSO_4_, 27 mM KCl, 1.5% Oxoid peptone, 50 mM Tris/HCl pH 7.5). For minimal inhibitory concentration (MIC) experiments, the cells were grown in complex medium containing the respective metal ion concentration adjusted using stock solutions of CuSO_4_ (50 mM), NiSO_4_ (100 mM), or ZnSO_4_ (100 mM). Planktonic cells were grown in cultures shaking at 180 rpm with a start optical density (OD_600_) of 0.02. For metal resistance experiments, metal solutions (final concentrations: 5, 8, and 10 mM copper; 15, 20, and 40 mM nickel; 40, 80, and 100 mM zinc) were added at OD_600_ 0.3 and the cells were cultivated for another 24 h. Adherent cells were grown in large Petri dishes (150/20 mm, Sarstedt) without shaking. After 12 days of cultivation, the medium was replaced by complex medium containing the respective metal ions in the final concentrations as above and the biofilms were cultivated for another 24 h. For biofilm isolation, the supernatant was removed and the dishes were washed three times with 10 mL salt solution (4.3 M NaCl, 81 mM MgSO_4_, 27 mM KCl, 50 mM Tris/HCl pH 7.5). Biofilms were scraped from the surface using a spatula.

### Fluorescence-Based Adhesion Assay

Adherence in the presence of metal ions was investigated using a fluorescence-based adhesion assay. *H. salinarum* R1 was cultivated in 24-well microtiter plates (2 mL complex medium containing the respective metal ion concentration per cavity) at 37°C. After 13 days of cultivation, acridine orange (Merck KGaA) was added to a final concentration of 1 μg/mL to each cavity and incubated for 15 min in the dark. Supernatants were discarded and the wells were washed three times with 1 mL salt solution. Fluorescence signals of adherent cells were detected at 473 nm (blue filter) using a PhosphorImager (FLA 5000, Fujifilm) and Image Reader FLA 5000 Series software. For quantitative analysis of the fluorescence signals (Light Absorbing Units per mm^2^) the software Image Gauge V4.23 was used. Background signals of controls containing only the respective growth medium were subtracted from the sample signals. For the calculation of the mean and standard deviation at least four independent samples of cells were used in each case. For comparison of the relative adhesion of metal-treated cells, the mean value of the signals of untreated samples was set to 100%. The significance (*P*-value) of the fluorescence signal of metal treated samples compared to the control sample was calculated by an unpaired, two-tailed *t*-test.

### Propidium Monoazide Treatment

For the treatment with propidium monoazide (PMA), *H. salinarum* spheroplasts were generated by resuspending *H. salinarum* cell sediments in 0.5 mL spheroplasting- (SPH) solution 1 (2 M NaCl, 25 mM KCl, 15% sucrose) plus 5 μL (planktonic cells) or 20 μL (biofilm cells) SPH-solution 2 (2 M NaCl, 25 mM KCl, 15% sucrose, 0.5 M EDTA). After 10 min, the formation of spheroplasts was analyzed by light microscopy. In each case, 0.25 mL were transferred to a 1.5 mL tube and PMA was added to a final concentration of 50 μM (planktonic cells) or 100 μM (biofilm cells). Following a 10-min incubation in the dark with occasional shaking, PMA-treated and untreated samples were transferred to a 24-well microtiter plate and light exposed using blue wavelength light-emitting diodes (LED, 470 nm, 16.000 mcd) inside a closed box equipped with a light-reflective foil. The 24-well microtiter plate was placed 0.5 cm from the light source and cooled on ice to avoid excessive heating. During the 40-min exposure to light the microtiter plate was shaken gently.

### Cell Lysis and Quantitative Real-Time Polymerase Chain Reaction

*Halobacterium salinarum* DNA was extracted by osmotic lysis of the cells. For this, 5 μL of the PMA-treated samples were mixed with 45 μL ddH_2_O on a vortex mixer for 1 min. The resulting cell lysate was used for the DNA amplifications. The qPCR reactions were prepared in a final volume of 10 μL containing 5 μL of SensiFAST^TM^ SYBR Hi-ROX Kit (# BIO-92005, Bioline), 1 μL of *H. salinarum* cell lysate, 2 μL of 2 μM forward primer and 2 μL of 2 μM reverse primer (Table 1). The primers anneal to 16S ribosomal DNA and by PCR they give rise to a 579 bp fragment with a GC-content of 57% using the forward primer 16S-fwd-579 bp, or to a 103 bp fragment with a GC-content of 50% using the 16S-fwd-103 bp forward primer. The cycling parameters were 5 min at 95°C followed by 40 cycles of 15 s at 95°C, 45 s at 57°C and 80 s at 72°C. The analysis was performed using the StepOne^TM^ Real-Time PCR System (Applied Biosystems) and the StepOne^TM^ software v2.0. Each DNA sample was analyzed in triplicate. To calculate the PMA-induced inhibition of the amplification, *C*_T_ values from untreated DNA samples were subtracted from the respective *C*_T_ values of PMA-treated samples. Quantification of viable cells was based on a standard derived from Δ*C*_T_ values of different ratios of live and heat-killed (70°C, 10 min) *H. salinarum* cells.

**Table 1:** Oligonucleotides used for PMA-qPCR and qRT-PCR analyses.

Name	Oligonucleotide sequence (5′–3′)
16S-fwd-579bp	CGTTGAGTCCAATTAAACCGC
16S-fwd-103bp	TCCTTATTCGTGCACCACCT
16S-rev	GCGCGAAACCTTTACACTGT
OE4485R-qPCR-fwd	CGGCTCGCTGATTATTTCGC
OE4485R-qPCR-rev	CTCCATGACTCCCTCGATGC
OE4552F-qPCR-fwd	GTTCTCGACGTTCCTGGGA
OE4552F-qPCR-rev	CCAACAGGAGCGTGTCAAC
OE4555F-qPCR-fwd	CGACATCTTCAAGCGCACG
OE4555F-qPCR-rev	CTGTAGTACGCCGCGATGAT
OE4576F-qPCR-fwd	GACCTCGATCCGGACGTCTA
OE4576F-qPCR-rev	CGTTGGGTGAGGAAGTCGAT
OE5146R-qPCR-fwd	GAATGACACCACACCACGAC
OE5146R-qPCR-rev	CGCAAGGGAGATGTCTTCGA
OE5245F-qPCR-fwd	CCGCGAGCATATCACATACG
OE5245F-qPCR-rev	CGCATTCCTTTCGAGTAGCC
OE2042F-qPCR-fwd	GACACCTTCCTGTTCGGGG
OE2042F-qPCR-rev	GTTGCTGAGAAACGACCGC
OE2044F-qPCR-fwd	TTGTGAGGGCTGTGAAGACA
OE2044F-qPCR-rev	CTCCATCCACGGTCACAGT
OE3453R-qPCR-fwd	TCGGTTTCACACTCCACGT
OE3453R-qPCR-rev	GAGAGACGTACGCTGAGAGG
OE3668F-qPCR-fwd	GTACGACACGACCCTGGAG
OE3668F-qPCR-rev	GCTCCCCATCACGATCTTGT
OE4544R-qPCR-fwd	TCGGAGGATTTCGACCAGATC
OE4544R-qPCR-rev	GAGGTTCGGGAGTTCAACAAC
pilB1-qPCR-fwd	CCGGAAGTACAGCGAGGAG
pilB1-qPCR-rev	GGCTCTTGTTGGATTCGATG

### Confocal Laser Scanning Microscopy (CLSM)

To analyze the cell vitality of planktonic and biofilm cells, the cultures were stained with the membrane permeating and DNA intercalating dye acridine orange (Merck KGaA), and the membrane impermeable dye propidium iodide (Carl Roth). Sessile cells were grown in small Petri dishes (35/10 mm, Sarstedt) on polyethylene terephthalate surfaces in complex medium with a start OD_600_ of 0.003. After 12 days of cultivation, the medium was replaced by a metal-containing medium and the biofilms were cultivated for another 24 h. To visualize cells and EPS, biofilm samples were stained for 10 min with acridine orange at a final concentration of 10 μg/mL. Extracellular DNA and dead cells were stained with propidium iodide at a final concentration of 20 μg/mL for 10 min. Staining of glycosidic (α-mannopyranosyl and α-glucopyranosyl) residues in the biofilm matrix was done using Concanavalin A (ConA) Alexa Fluor^®^ 647 conjugates (Life Technologies) at a final concentration of 20 μg/mL for 15 min. Staining was performed in the dark followed by washing the biofilms three times with 2 mL salt solution to remove non-adherent cells. Microscopic analyses of the cell vitality and of the three-dimensional structure of the biofilms were performed using a confocal laser scanning microscope (TCS SP5 II, Leica Microsystems GmbH, Wetzlar, Germany) and the Leica Application Suite software. Image processing was done by use of ImageJ. Quantitative analysis of the biofilm mass was done by measuring the surface coverage of adherent cells of at least ten images using ImageJ.

### RNA Preparation

RNA isolation from planktonic cells exposed to metals was done by standard acid guanidinium thiocyanate-phenol-chloroform extraction (Chomczynski and Sacchi, 2006). Removal of genomic DNA was done by treatment with RNase-free DNase I (# EN0521, Thermo Fisher Scientific) treatment for 3 h at 37°C. Purified RNA was used for the generation of complementary DNA (cDNA).

### Quantitative Reverse Transcription Polymerase Chain Reaction (qRT-PCR)

For qRT-PCR, 2 μg of purified RNA was reversely transcribed into cDNA using Random Hexamer Primers (# SO142, Thermo Fisher Scientific) and RevertAid^TM^ Reverse Transcriptase (# EP0441, Thermo Fisher Scientific) in a total volume of 20 μL, according to the manufacturer’s protocol. qRT-PCR analysis was performed using the StepOnePlus^TM^ Real-Time PCR System in combination with the StepOnePlus^TM^ Software v2.3 (Applied Biosystems) and the SensiFast^TM^ SYBR Hi-ROX Kit (# BIO-92005, Bioline) according to the manufacturer’s protocol. The oligonucleotides used for qRT-PCR are listed in Table 1. Relative expression changes of the target gene in metal-treated cells compared to untreated cells was calculated using the *C*_T_-method. *C*_T_-values of the target genes were normalized to the housekeeping gene *rpoB1* (OE4741R) (Bleiholder et al., 2012). Each sample was measured in triplicates. The significances (*P*-values) of changes in the amount of gene expression were assessed by an unpaired, two-tailed *t*-test.

## Results

### Effect of Metal Ions on Planktonic Cells and Adhesion

**FIGURE 1:**
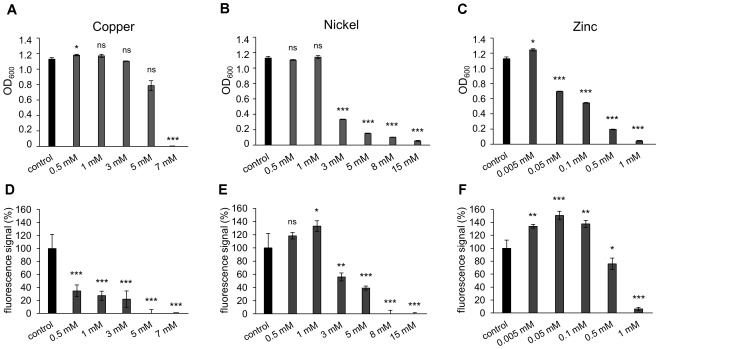
Effect of metal ions copper, nickel and zinc on planktonic growth and adhesion of *Halobacterium salinarum* R1. **(A–C)** Optical density of planktonic cultures of *H. salinarum* after growth in complex medium containing copper, nickel or zinc. Control, cultivation in complex medium. Diagrams show the final optical density at 600 nm (OD_600_) after 72 h cultivation. Data based on three independent cultures. **(D–F)** Fluorescence-based adhesion assay of *H. salinarum* after 13 days cultivation in complex medium containing copper, nickel or zinc ions. Control, cultivation in complex medium. Diagrams show the relative adhesion of the metal-treated cells in relation to untreated cells (set to 100%). Data based on four independent experiments. The significance of the results obtained with metal-treated samples compared to the control was assessed by *t*-test (ns, not significant, ^∗^ significant = *P* < 0.05, ^∗∗^ highly significant = *P* < 0.01, ^∗∗∗^ extremely significant = *P* < 0.001).

To test the growth of *H. salinarum* in the presence of the metal ions copper, nickel or zinc, the cell mass of planktonic cultures was quantified by measuring the optical density (OD_600_) after cultivation for 72 h in the stationary growth phase. For each metal, five or six concentrations up to the respective minimal inhibitory concentration (MIC; determined for planktonic cells) were added to the cultures at OD_600_ 0.02. As control, growth of cells was analyzed in media lacking the respective metals. In case of the addition of copper ions to planktonic cells, the cultures reached OD_600_ 1.1 up to 3 mM copper, indicating no difference compared to the control (Figure 1A). In the presence of 5 mM copper ions the OD_600_ value slightly decreased and 7 mM copper completely inhibited growth of the planktonic cells (Figure 1A). In the case of nickel ions, growth was not affected up to 1 mM (Figure 1B). However, increased nickel concentrations (3–15 mM) resulted in a significant decrease of the planktonic growth (Figure 1B). In case of zinc ions, cultivation in the presence of 0.005 mM zinc increased the amount of cells compared to the control, whereas 0.05 mM and higher zinc concentrations decreased the cell growth (Figure 1C). Almost no growth was observed in cultures containing 1 mM zinc. In conclusion, the three different metal ions led to metal-specific behaviors of planktonic cultures (Table 2). The OD value reached after 72 h was not altered even after 13 days of cultivation for each metal (data not shown).

**Table 2:** Metal concentrations causing inhibition of planktonic growth, cell attachment and killing of planktonic cells compared to biofilms.

	Copper	Nickel	Zinc
**Inhibition**			
Planktonicgrowth	≥7 mM	≥15 mM	≥1 mM
Adhesion and biofilm formation	≥5 mM	≥8 mM	≥1 mM
**Killing of 100% cells**			
Planktonic cultures	10 mM	>40 mM	100 mM
Biofilms	>10 mM	>40 mM	>100 mM

Similar experiments were performed with cells adhering to plastic surfaces and forming biofilms. Surface adhesion in the presence of metal ions was quantified with a fluorescence-based adhesion assay after 13 days of cultivation. In the presence of up to 3 mM copper ions adhesion was significantly reduced to approximately 30% compared to the control and completely inhibited at copper concentrations of 5 and 7 mM (Figure 1D). These results suggested a stronger inhibiting effect of copper ions on surface adhesion and biofilm formation compared to the planktonic growth (Figures 1A,D). In the presence of nickel ions cell adhesion was increased up to 1 mM (Figure 1E). Higher nickel concentrations led to a decreased adhesion, and no adhesion was observed at 8 and 15 mM nickel. Thus, the effect of nickel ions was similar on biofilms compared to planktonic cells (Figures 1B,E). In the presence of zinc ions, adhesion of cells increased up to 150% at 0.005 to 0.1 mM zinc, while 0.5 mM and higher concentrations strongly inhibited adhesion (Figure 1F). Comparing the influence of zinc ions on planktonic growth *versus* surface adhesion, an increased adhesion and biofilm formation was observed at concentrations up to 0.1 mM zinc, whereas the growth of the planktonic culture was already reduced at these concentrations (Figures 1C,F). The MIC for each metal was determined by colony count on agar plates and underlined the results determined by OD measurements (data not shown). Overall, different MICs were determined for planktonic cells with the respective metal tested, i.e., 7 mM for copper, 15 mM for nickel and 1 mM for zinc, whereas adhesion of cells was inhibited at lower concentrations, i.e., 5 mM copper, 8 mM nickel and 1 mM zinc (Table 2).

### Adjustment of PMA-qPCR to Determine the Cell Vitality in Haloarchaeal Cells

Propidium monoazide (PMA) is a DNA-intercalating fluorescence dye targeting DNA of membrane-disrupted, dead cells only. After photoactivation, PMA binds covalently to DNA and inhibits DNA amplification in a subsequent PCR assay. Using the *C*_T_-values for defined mixtures of live and dead cells, the amount of dead cells after PMA treatment can be determined. For optimal quantification, the difference between *C*_T_ values of live and dead cells should be at least 10 cycles. So far, this method was used to analyze bacteria, but it is affected by high salt concentrations (Barth et al., 2012). To quantify the effect of metals on haloarchaea, the method needs an adjustment to the high internal salt concentrations of up to 4–5 M KCl. To adjust the method, the suppression of the amplification of DNA by PMA was tested with isolated genomic DNA in the presence of 4.3 M (25%) NaCl in comparison to DNA in water. DNA of *H. salinarum* was incubated in water containing 100 μM PMA for 10 min and light exposed before amplification of a 103 bp sub-fragment of the gene encoding 16S rRNA. In addition, samples not exposed to PMA were tested as control by qPCR (Supplementary Figure S1A). In the presence of 4.3 M NaCl, the *C*_T_ of untreated DNA was around 20, while PMA-exposed DNA resulted in a *C*_T_ of 25, indicative of a weak suppression of amplification (Δ*C*_T_ 5). However, in the absence of NaCl the Δ*C*_T_ of untreated DNA compared to PMA-exposed DNA was approximately 13, indicating a strong suppression of amplification (Supplementary Figure S1A). To investigate the effect of PMA treatment on *H. salinarum* cells, live and heat-killed (70°C, 10 min) cells were tested in 4.3 M NaCl solution and exposed to PMA as described. No suppression was detected in case of PMA-exposed live cells, whereas PMA exposure of dead cells resulted in a Δ*C*_T_ around 5, indicating a weak suppression of amplification by qPCR (Supplementary Figure S1B). Several modifications of the standard protocol were performed to increase the suppression of DNA amplification in dead *H. salinarum* cells. Firstly, the NaCl concentration was reduced to 2 M (11.6%), the lowest tolerable concentration for *H. salinarum*, in SPH-solution 1 containing 25 mM KCl and 15% sucrose to stabilize the cells. A reduction to 2 M NaCl with 15% sucrose in combination with EDTA treatment showed no effect on cell damage (data not shown). To increase the permeation of PMA to dead cells, the S-layer protein of *H. salinarum* was disaggregated by the addition of EDTA to a final concentration of 5 mM. The formation of spheroplasts was controlled by light microscopy where the rod-shaped *H. salinarum* cells round up into spheres. In addition, a longer PCR amplicon (579 bp) was used to increase the probability of the PMA induced interference with the elongation process. This effect has been shown in studies using PMA-qPCR applied to *Vibrio anguillarum* and *Flavobacterium psychrophilum* (Contreras et al., 2011). Both lengths of the amplicons (103 and 579 bp 16S rDNA fragments) were tested with *H. salinarum* R1. In case of the shorter DNA fragment, the pre-treatment of dead cells with EDTA, followed by the exposure to PMA resulted in a Δ*C*_T_ 3, indicating a weak suppression of amplification (Supplementary Figure S1C). In contrast, amplification of the longer 579 bp DNA fragment resulted in a Δ*C*_T_ > 10, indicative of a strong suppression of the amplification by PMA treatment (Supplementary Figure S1C). PMA-exposure of dead cells without previous EDTA-treatment and amplification of the longer DNA fragment resulted in a Δ*C*_T_ 6 (Supplementary Figure S1C). In conclusion, pre-treatment of cells with EDTA in combination with an amplification of a larger PCR amplicon increased the suppression of DNA amplification of dead cells, whereas the amplification of DNA in live cells was not affected. To achieve similar Δ*C*_T_-values for *H. salinarum* cells grown as biofilm, an increase of the EDTA concentration to 20 mM was required to form spheroplasts as checked microscopically. In addition, different PMA concentrations were tested. A final concentration of 100 μM PMA ensured the most effective inhibition of DNA amplification in biofilm cells (data not shown).

The number of dead cells was calculated using a standard obtained with cell samples containing defined amounts of heat-killed *H. salinarum* cells. Mixtures with rising amounts of heat-killed cells (0, 25, 50, 75, 90, 99, 99.9, and 100%) were prepared, pre-treated with EDTA and exposed to PMA, followed by the amplification of a 579 bp DNA fragment (Supplementary Figure S2A). A linear dependence was found in the range between 0 and 75% dead cells, while 90% and higher amounts of dead cells resulted in larger Δ*C*_T_ values (Supplementary Figure S2A). To verify the ratios between live and dead cells of the generated PMA-qPCR standard, identical mixtures of the cells were live/dead stained with acridine orange (AO) and propidium iodide (PI) and investigated by fluorescence microscopy (Supplementary Figure S2B). AO permeates in cells and stains DNA of live and dead cells (green signal), while PI is not permeant to live cells and will detect dead cells only (red signal). Live *H. salinarum* cells were rod-shaped and about 3–5 μm in length, while heat-killed cells had a spherical morphology with a diameter of about 2 μm (Supplementary Figure S2B). For each ratio, at least 500 cells were counted, and the number of dead cells (PI stained) was quantified and calculated as percentage of the total cell number (Supplementary Figure S2C). A linear correlation was observed between the increasing amount of dead cells and an increasing number of spherical, PI stained cells (Supplementary Figure S2C). Also, a decrease in CFU/mL was observed with an increasing amount of dead cells in additional cultivation tests on agar plates, verifying the ratios between live and dead cells of the PMA-qPCR standard generated (data not shown).

### Cell Vitality of Metal Treated *H. salinarum* Cells

**FIGURE 2:**
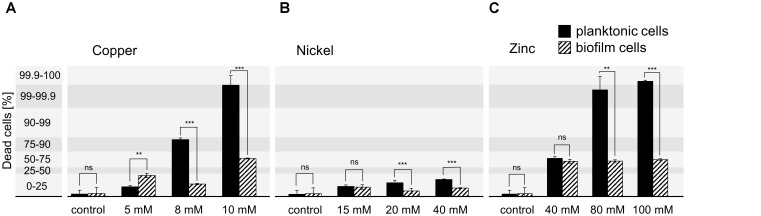
Quantification of dead *H. salinarum* cells after metal treatment. Planktonic cells (OD_600_ 0.3) and 13 days biofilms were incubated with growth inhibiting concentrations of **(A)** copper, **(B)** nickel, or **(C)** zinc ions for 24 h. The percentage of dead cells was quantified by PMA-qPCR and compared to standard Δ*C*_T_ values corresponding to defined amounts of dead cells. Untreated cells were used as control. The significance of the data from planktonic compared to biofilm samples was assessed by *t*-test (ns, not significant, ^∗^ significant = *P* < 0.05, ^∗∗^ highly significant = *P* < 0.01, ^∗∗∗^ extremely significant = *P* < 0.001). The effect of the highest tested heavy metal concentrations was also investigated by confocal microscopy (Figures 3, 4).

The optimized PMA-qPCR was used to quantify the amount of dead cells in planktonic and biofilm samples upon metal exposure. The respective metal concentrations were strongly increased above the MIC values determined for planktonic cells to achieve cell death in planktonic and biofilm cultures as well. In case of copper ions, concentrations of 5, 8, and 10 mM were tested, whereas the effect of nickel ions was tested at 15, 20, and 40 mM and zinc ions at 40, 80, and 100 mM. Planktonic cells were grown to exponential growth phase (OD_600_ 0.3) in medium without metal ions and were then exposed to the respective metal concentration for 24 h. In addition, *H. salinarum* biofilms were grown in medium for 13 days before the supernatant was replaced by medium supplemented with metal ions. The biofilms were then incubated for another 24 h. After metal exposure, planktonic cells and biofilms were pre-treated with EDTA and incubated with PMA at the respective optimized conditions before the DNA was amplified by qPCR. The resulting Δ*C*_T_ determined for each condition was compared to the PMA-qPCR standard Δ*C*_T_ values corresponding to defined amounts of dead cells (Figure 2). In case of planktonic cells exposed to copper, up to 90% and up to 100% dead cells were observed in the presence of 8 and 10 mM copper ions. In contrast, biofilms showed up to 25% dead cells at 8 mM and up to 75% dead cells at 10 mM copper ions (Figure 2A). In the presence of 5 mM copper ions, cells grown in liquid cultures or as biofilm showed similar amounts of dead cells (Figure 2A). Thus, cells in biofilms were relatively well protected at higher copper concentrations. In case of nickel ion exposure, up to 25% dead cells were observed in the presence of 15, 20, and 40 mM (maximal concentration at physiological pH-value), in both planktonic and biofilm samples. However, the amount of dead cells was slightly higher for planktonic cells compared to biofilms (Figure 2B). In the case of zinc, treatment of planktonic cells with 40 mM of zinc ions yielded 50–75% dead cells, whereas 80 and 100 mM zinc killed almost all of the planktonic cells (Figure 2C). In contrast, cells grown as biofilm showed a constant amount of dead cells up to 75% irrespective of the zinc concentration (Figure 2C). Compared to the MIC determined for each metal, the concentration used to kill 100% of the planktonic cells was increased in the case of copper from 7 mM (MIC) to 10 mM and in the case of zinc from 1 mM (MIC) to 100 mM. However, in the case of nickel, cells were able to cope with even 40 mM, since only 25% of the planktonic or biofilm cells were dead under these conditions (Table 2).

### Effect of Metal Ions on Cell Morphology and Architecture of Biofilms

**FIGURE 3:**
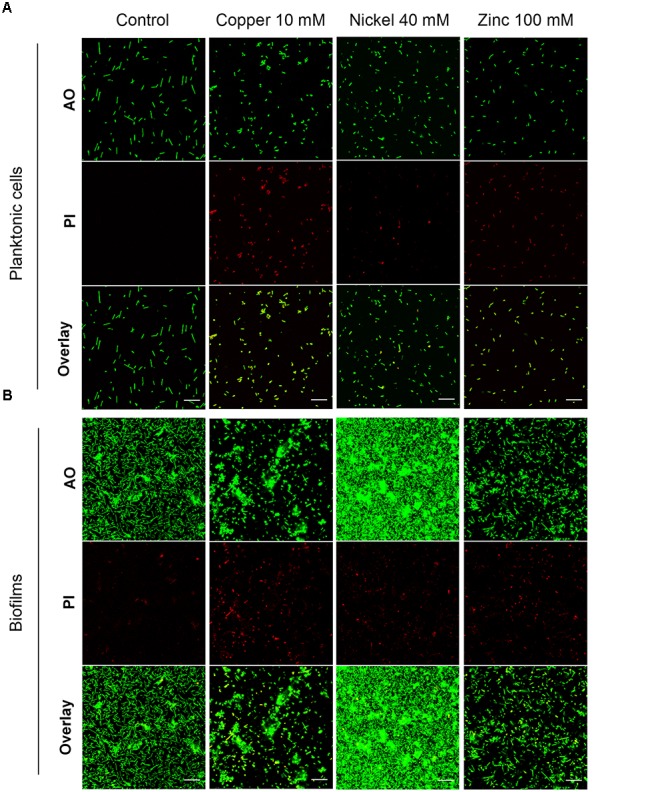
Effect of 24 h exposure to metal ions on *H. salinarum* planktonic cells and mature biofilms. Before staining planktonic cells **(A)** were metal treated at OD_600_ 0.3, biofilms **(B)** were exposed to metals after 12 days of growth in complex medium. The images show the biofilms at the bottom of the wells. Live/dead staining was done using acridine orange (AO) to stain intra- and extracellular nucleic acids, and propidium iodide (PI) staining eDNA and dead cells. Scale bars equal 20 μm in each case.

The highest metal concentration tested in each case, 10 mM copper, 40 mM nickel or 100 mM zinc ions, was used to investigate planktonic cells and mature biofilms by confocal laser scanning microscopy. Planktonic cells were grown to OD_600_ 0.3 followed by a 24 h exposure to the respective concentrations of the metal ions. Live/dead staining of the cells was done with acridine orange (AO, live cells) and propidium iodide (PI; dead cells). In the control sample of the planktonic culture all of the cells were alive and exhibited a rod-shaped morphology up to a length of 5 μm (Figure 3A, control). Exposure of planktonic cells to 10 mM copper ions resulted in 100% dead cells. The morphology was altered to short rods (2 μm) or to spheres in most cases (Figure 3A, copper). In contrast, 40 mM nickel ions yielded a small amount of dead cells with spherical shape, while most of the cells were alive and rod-shaped (Figure 3A, nickel). In the presence of 100 mM zinc ions, rod-shaped planktonic cells were observed; however, all of the cells were dead (Figure 3A, zinc).

Biofilms were grown in small Petri dishes on polyethylene terephthalate surfaces with complex medium for 12 days followed by a 24 h exposure to inhibitory concentrations of the metal ions. Live/dead staining of the untreated control samples showed a small amount of PI-stained (dead) cells and eDNA, but most of the cells were alive, rod-shaped and attached to the surface forming a monolayer of cells and several small microcolonies (Figure 3B, control). Treatment of biofilms with 10 mM copper ions resulted in a decreased number of single cells on the surface and the formation of large microcolonies. Live/dead staining showed spherical dead cells predominantly in regions of single adherent cells, but the cells in microcolonies were alive. This result suggested that cell survival was supported in the microcolonies surrounded by EPS (Figure 3B, copper). Exposure to 40 mM nickel ions resulted in an extensive multi-layered cell adhesion with densely packed cell aggregates (Figure 3B, nickel). Only a few dead cells were observed on the colonized surface. The effect of 100 mM zinc ions did not show significant changes of the biofilm architecture compared to the control sample, however, a large fraction of sessile cells was dead (Figure 3B, zinc).

**FIGURE 4:**
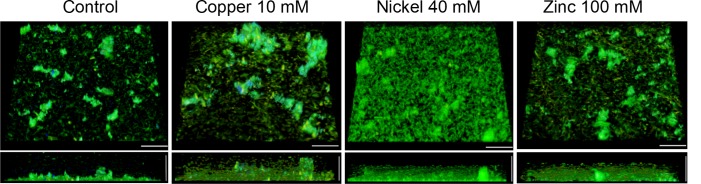
Confocal laser scanning micrographs (CLSM) of *H. salinarum* biofilm structures after 24 h exposure to metal ions. Tilted top view **(top)** and side view **(bottom)** of biofilms grown for 12 days in complex medium and after different metal treatments for 24 h. Genomic DNA was stained with acridine orange (green), whereas eDNA and dead cells were stained with propidium iodide (red). Glycoconjugates (α-mannopyranosyl and α-glucopyranosyl residues) were stained using Concanavalin A (ConA) Alexa Fluor^®^ 647 (blue). Scale bars equal 25 μm in each case.

The effects of the highest tested metal concentration on the three-dimensional biofilm structure and the composition and localization of the EPS were also inspected by confocal laser scanning microscopy. In addition to AO and PI staining, biofilms were stained with Concanavalin A (ConA) Alexa Fluor^®^ 647 conjugates to detect glycosidic residues in the biofilm matrix. Three-dimensional images of untreated biofilms showed monolayers of cells covering 17.6 ± 3.1% of the surface and microcolonies about 20 μm in height, containing small amounts of eDNA and glycoconjugates in the aggregates (Figure 4). Treatment with 10 mM copper ions resulted in a decrease of single sessile cells covering 7.9 ± 2.2% of the surface and the formation of large cell aggregates with a high amount of eDNA/dead cells and glycoconjugates in the upper parts of the aggregates (Figure 4). Nickel-treated (40 mM) biofilms showed a significantly higher number of adherent cells on the surface constituting a dense multilayer with frequently appearing microcolonies around 20 μm in width. The dense biofilms covered 64.1 ± 6.7% of the surface. In the presence of 100 mM zinc ions no significant changes of the biofilm architecture or of the amount of sessile cells covering 13.5 ± 2.6% of the surface were observed compared to the untreated biofilm (Figure 4).

### Expression of Selected Genes in Metal-Treated and Untreated Planktonic Cells

To determine an effect at the molecular level, the gene expression of putative transport systems in *H. salinarum* R1 and of flagella- and pili genes was investigated by qRT-PCR. The expression of the flagella and pili assembly/motor-ATPase encoding genes (*flaI*, *pilB1*, and *pilB2*) was not affected by metal-treatment (data not shown). The relative expression of genes encoding ATP binding cassette (ABC) transporters, metal transporters as well as two genes encoding universal stress proteins harboring on UspA domain (OE3368F and OE4544R) was quantified (Table 3).

**Table 3:** Target genes for gene expression studies in metal treated and untreated planktonic cells.

Gene name	Gene description^1^	Protein name^2^	Fold change in gene expression^3^		
	
ABC transporter associated proteins			Copper	Nickel	Zinc
OE4485R *pstS1*	Phosphate ABC transporter substrate-binding protein	ABC-type transport system periplasmic substrate-binding protein (probable substrate phosphate)	-126.6	-13.7	-1.2
OE4552F *dppB2*	ABC transporter permease	ABC-type transport system permease protein (probable substrate dipeptide/oligopeptide)	-4	3.5	-6.2
OE4555F *dppC2*	ABC transporter permease	ABC-type transport system permease protein (probable substrate dipeptide/oligopeptide)	-2.8	1.4	-2.1
OE4576F	Hypothetical protein	ABC-type transport system periplasmic substrate-binding protein	21.8	2.5	6.0
OE5146R *znuC*	Metal ABC transporter ATP-binding protein of Znu ABC transporter	ABC-type transport system ATP-binding protein (probable substrate zinc)	-4.2	38	18.3
OE5245F	ABC transporter ATP-binding protein	ABC-type transport system ATP-binding protein	-3.3	-2.3	-14.3
**Metal transporter associated proteins**					
OE2042F *copA*	Cu^2+^-exporting ATPase	P-type transport ATPase (probable substrate copper/metal cation)	15.3	21.4	667.6
OE2044F	Copper chaperone	HMA domain protein	1.5	2.4	6.0
OE3453R	ZIP family metal transporter	Uncharacterized protein	-19.2	7.3	3.6
**UspA domain proteins**					
OE3668F	Universal stress protein	UspA domain protein	-1.8	12.1	3.1
OE4544R	Universal stress protein	UspA domain protein	3.4	19.5	2.9

*^1^According to NCBI database (https://www.ncbi.nlm.nih.gov/). ^2^According to UniProtKB database (https://www.uniprot.org/). ^*3*^Compared to untreated cells*.

For the qRT-PCR analysis, planktonic cells were treated with the metals copper, nickel and zinc in growth-inhibiting concentrations of 5 mM Cu, 15 mM Ni and 1 mM Zn. Total RNA was isolated after 24 h growth of metal treated and untreated cells. For quantitative analysis, the *C*_T_ values were normalized to the “housekeeping” gene *rpoB1* (DNA-directed RNA polymerase subunit B’) and the metal-treated samples were compared to the control, respectively. While the analysis yielded qRT-PCR products for planktonic cells, a similar analysis using metal-treated biofilm cells was impossible. No products were observed in qRT-PCR using these cells. A reason for the latter result might be that the metals attached to the EPS are not washed off and disturb the activity of enzymes applied for qRT-PCR. Several protocol alterations such as separating the biofilm matrix from the cells did not improve the method.

**FIGURE 5:**
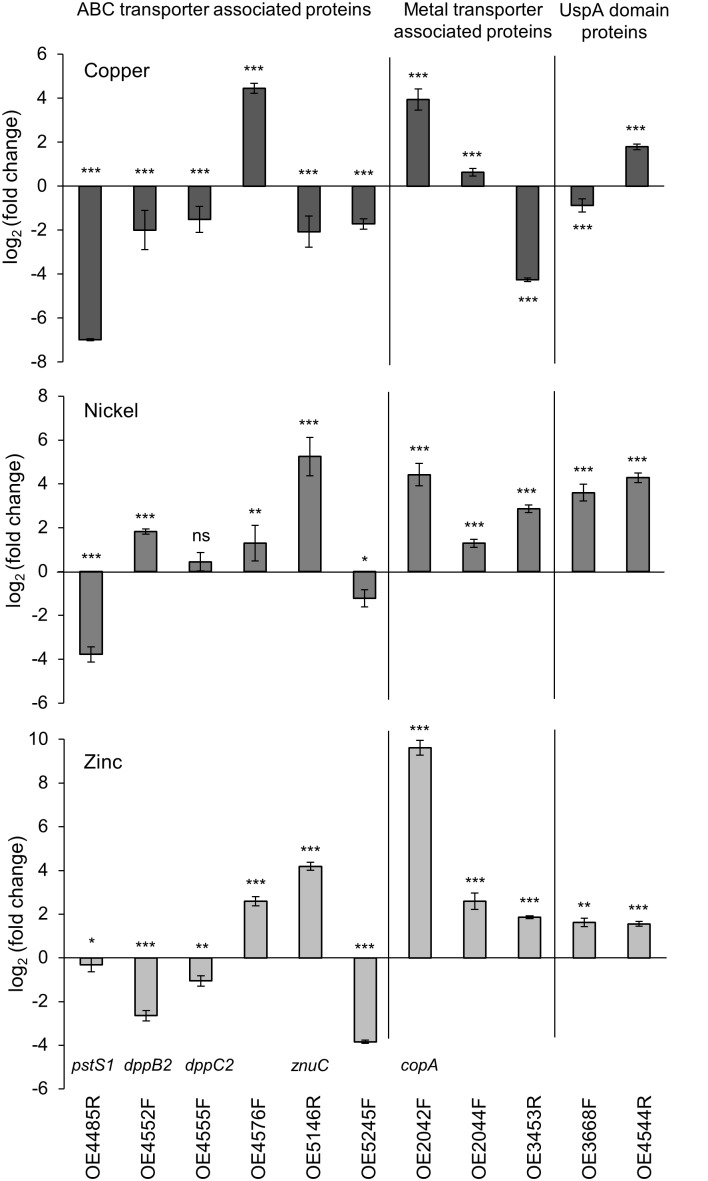
Comparative qRT-PCR analyses of metal treated and untreated planktonic cells. Gene expression of several ABC transporter- and metal transporter encoding genes as well as two UspA domain genes was relative quantified. Relative quantification in **(A)** copper-treated, **(B)** nickel-treated, and **(C)** zinc-treated cells compared to untreated cells. *C*_T_-values of the target genes were normalized to the housekeeping gene *rpoB1*. Significance of changes in the amount of transcripts was assessed by *t*-test (ns, not significant, ^∗^ significant = *P* < 0.05, ^∗∗^ highly significant = *P* < 0.01, ^∗∗∗^ extremely significant = *P* < 0.001). For description of genes and the corresponding fold change in gene expression see Table 3.

The results of planktonic cells are presented in Figure 5 and Table 3. In case of the copper-treated cells, the relative expressions of *pstS1*, of the two ABC transporter permease genes *dppB2* and *dppC2*, of *znuC* encoding a subunit of the ZnuABC transporter and of OE5245F was decreased (Figure 5A). In contrast, OE4576F encoding a hypothetical ABC transport system showed a 22-fold enhanced expression. Among the genes encoding metal transporter associated proteins, the Cu^2+^-exporting ATPase *copA* and the corresponding copper chaperone OE2044F showed an enhanced expression, whereas the expression of the ZIP family metal transporter OE3453R was decreased. The expression of the two universal stress proteins was decreased in case of OE3668F and increased in case of OE4544R (Figure 5A). The nickel-treated cells yielded different results for many of these genes. The expression of *pstS1* and OE5245F was decreased similar to copper treatment, but the remaining genes encoding ABC transporter-associated proteins showed an enhanced expression compared to untreated cells (Figure 5B). The expression of the metal transporter associated proteins *copA*, OE2044F, and of OE3453R was enhanced, as were the expression of the two universal stress proteins OE3668F and OE4544R (Figure 5B). In the case of zinc-treated cells the expression of *pstS1*, *dppB*2, *dppC2*, and OE5245F was decreased, while the remaining genes encoding ABC transporter-associated proteins showed an enhanced expression with a fold change up to 668 in the case of the Cu^2+^-exporting ATPase *copA* (Figure 5C). Also, the remaining genes encoding metal transporter-associated proteins and universal stress proteins showed an enhanced expression in response to zinc treatment (Figure 5C).

In conclusion, seven of the eleven investigated genes showed a decreased expression in copper-treated cells, whereas in zinc-treated cells the expression of four genes- and in nickel-treated cells the expression of only two genes was decreased. Among the eleven genes tested, six genes showed a similar reaction in expression in case of all three metal treatments, while the expression of the other five genes was altered differently. These results suggested metal-specific response mechanisms in *H. salinarum*.

## Discussion

The effect of heavy metal ions on the survival of haloarchaea has been studied to some extend (Bini, 2010; Voica et al., 2016; Chandrangsu et al., 2017), but the effect of metal ions was not yet investigated with regard to adhesion and biofilm formation. We determined the effects of copper, nickel, and zinc ions on growth of the haloarchaeon *H. salinarum* R1 and quantified the cell vitality of planktonic cells and biofilms. Microscopic techniques visualized alterations of the biofilm architecture after metal treatment and qRT-PCR analysis demonstrated alterations in the expression of transport systems and stress proteins in *H. salinarum*.

### An Adjusted PMA-qPCR Was Successfully Applied to Haloarchaea

To quantify the cell vitality apart from microscopic techniques, a PCR method based on propidium monoazide (PMA), a photoreactive dye intercalating into the DNA of dead cells and inhibiting its amplification after photoactivation, was adjusted for haloarchaea. A previous study demonstrated that the procedure does not function in the presence of more than 5% sodium chloride (Barth et al., 2012). This result agrees with the data presented here, since PMA inhibited the DNA amplification only weakly in the presence of 4.3 M (25%) NaCl, compared to 100% inhibition of the PCR when DNA is present in water. Adjusting the salt concentration to 2 M (11.6%), i.e., the lowest tolerable concentration for *H. salinarum*, led to a stronger inhibition of DNA amplification after PMA treatment. In addition, the cells were pre-treated with EDTA to obtain spheroplasts, and this procedure was also helpful to increase the DNA amplification. PMA is able to permeate the cell membrane of dead spheroplasts more effectively and inhibits DNA amplification by PCR. Also, the amplicon size affected the PMA-qPCR. Recent studies on several bacteria show that longer amplicons improved the inhibition of DNA amplification in PMA-qPCR (Contreras et al., 2011; Banihashemi et al., 2012). In the case of *H. salinarum*, the longer amplicon size increased the probability that PMA bound to DNA and strongly inhibited the DNA amplification. The adapted procedure allowed to quantify the amount of dead cells after metal exposure of *H. salinarum* planktonic cells and biofilms.

### The Effects of Copper, Nickel and Zinc on Planktonic Growth and Adhesion Are Metal-Specific

The heavy metals ions copper, nickel and zinc are essential trace elements in development and growth of all living organisms. Copper and nickel are mainly required as redox cofactors in the catalytic centers of enzymes, like the Cu-containing cytochrome *c* oxidase or the Ni-superoxide dismutase (Macomber and Hausinger, 2011; Chasapis et al., 2017). In addition, zinc is able to stabilize membranes and macromolecules and is a common constituent of DNA binding proteins (Shankar and Prasad, 1998; Choudhury and Srivastava, 2001). However, excessive metal concentrations lead to cell death. The minimal inhibitory concentrations (MICs) of these metals were determined for *H. salinarum* after 72 h cultivation. A MIC of 7 mM was found for copper, 15 mM for nickel and 1 mM for zinc ions, showing that different amounts of metals are tolerated. MIC studies in bacteria determined much lower values, i.e., 1.5–1.6 mM for copper and 1.5 mM for zinc in *Pseudomonas* and *Proteus*, and the MIC in Gram-positive bacteria such as *Bacillus* and *Staphylococcus* is even lower and at 0.2 and 0.5 mM (Hassen et al., 1998). The higher resistance of Gram-negative bacteria is attributed to their outer membrane. As determined in our study, haloarchaea were able to grow in the presence of higher metal concentrations. This was also shown in a previous study where MIC of different metal ions was investigated for 13 different haloarchaeal strains (Nieto et al., 1987). The genera *Haloferax*, *Halobacterium*, *Halorubrum*, and *Haloarcula* were investigated using an agar dilution method, and growth was not inhibited even at the highest tested concentration of 2.5 mM in case of nickel and zinc ions (Nieto et al., 1987). In another study, growth of *Halococcus* and *Haloferax* was observed in the presence of up to 2 mM zinc and zinc oxide nanoparticles (Salgaonkar et al., 2015). At salt concentrations of 15% (w/v) NaCl, the toxicity of heavy metals was increased compared to cells grown at high salt concentrations [25% (w/v) NaCl]. This effect could be related to the physiological state of the cells and/or indicates an influence of salts in the culture medium affecting the availability of metals to the cells (Nieto et al., 1987). Possible interactions between free metal ions and salts in the culture medium might be a reason for the increased metal tolerance of haloarchaea compared to non-halophilic species and could be the reason for the increased tolerance and resistance of haloarchaea in metal polluted environments.

So far, the influence of metals on haloarchaeal species was only studied with planktonic cells, whereas studies on cell adhesion in the presence of metal ions were lacking. We determined the respective concentrations of metal ions fully inhibiting cell adhesion, i.e., 5 mM for copper, 8 mM for nickel and 1 mM for zinc (Table 2). The values were lower than the MICs determined for respective planktonic cultures, suggesting a strong effect of the metal ions on cell adhesion. In the case of copper, cell adhesion was also reduced with subinhibitory concentrations, whereas the lowest concentrations of nickel and zinc used here even induced the cell adhesion and biofilm formation. The hyperthermophilic euryarchaeon *Archaeoglobus fulgidus* also showed an induced biofilm formation when exposed to 3 ppm (50 μM) copper or nickel (Lapaglia and Hartzell, 1997). In studies with microfluid chambers, copper concentrations below the MIC inhibited cell aggregation and prevented the formation of biofilms in *Xylella fastidiosa* (Cobine et al., 2013). In the case of *Staphylococcus aureus*, the presence of copper ions led to a repression of genes involved in the biofilm formation (Baker et al., 2010). These results demonstrate that the effects of heavy metal ions are species- and metal-specific and either lead to an inhibition or an enhanced adhesion and biofilm formation.

### Mature Biofilms Exhibit Increased Cell Survival Rates Compared to Planktonic Cells

The PMA-qPCR assay developed here enabled us to investigate the cell vitality of *H. salinarum* when planktonic cells or biofilms were exposed to metals. Different tolerance ranges were observed for each metal ion with planktonic cells. An increase to 10 mM copper resulted in 100% dead cells, whereas a larger increase from 1 mM (MIC) to 100 mM zinc was required to kill all of the planktonic cells. In the presence of nickel ions, <25% dead cells were obtained with any concentration tested. Higher concentrations than 40 mM nickel were not possible due to a shift to the acidic pH range. Comparing the MICs of all three metals with the results of the cell vitality experiments, it becomes clear that the respective MIC inhibits growth, but the cells are still alive and presumably in a non-growing or dormancy state. In bacteria two well-defined dormancy states have been described: the viable but non-culturable (VBNC) state and the presence of persister cells. Cells enter the VBNC state in response to environmental stress, such as a change of growth temperature, osmotic pressure or the oxygen concentration, and their metabolic activity is very low. However, the cells have the ability to become culturable once resuscitated (Oliver, 2005). Persister cells are described as a small subpopulation of slow or non-growing cells usually occurring in dense populations in the stationary growth phase or in biofilms (Lewis, 2000, 2001). Whether persisters or cells in VBNC state occur in archaeal cultures or biofilms is still unknown. We demonstrated that cells living as biofilm were less sensitive to metal ions compared to planktonic cells. While 10 mM copper and 100 mM zinc caused 100% of dead planktonic cells, similar amounts of these metals yielded 50% or 75% dead cells in biofilms, indicating an increased resistance or time-dependent tolerance of cells in biofilms. Biofilm-mediated resistance is based on different factors, namely a lower metabolic activity of cells in biofilms compared to planktonic cells and the absorbance of such toxic substances by cells located on the outside or by the extracellular polymeric substances (EPS). EPS components provide a high concentration of charged functional groups, like carboxyl-, hydroxyl-, phosphoryl- and amino groups that are able to bind and immobilize metal ions (Hullebusch et al., 2003). The number of electrostatic binding sites in the EPS matrix is 20- to 30-fold increased compared to the bacterial surface as shown in hydrogen-producing sludge and sulfate-reducing biofilms, and decreases or even prevents the permeation of metals (Liu and Fang, 2002). It is likely that the EPS of haloarchaeal biofilms also contributed to the increased survival rates in biofilms.

### Alterations of the Biofilm Structure Are Metal-Specific

Metal treatment also affected the architecture of *H. salinarum* biofilms. Exposure to 10 mM copper resulted in a decrease of single adherent cells and the formation of large cell aggregates with 50–75% dead cells. The cells alive persisted inside large cell aggregates that might produce increased amounts of EPS components for cell protection. Studies on the metabolism of planktonic cells and biofilms of *Pseudomonas aeruginosa* exposed to copper ions showed an induction of genes involved in exopolysaccharide metabolism, suggesting a protective response to metal stress (Booth et al., 2011). Genes involved in EPS metabolism in haloarchaea are still unknown. The exposure to nickel ions at growth inhibiting concentrations resulted in a massive increase of adherent cells forming dense cell layers and cell aggregates at the surface, similar to biofilm formation of *Escherichia coli* induced by sub-inhibitory concentrations of nickel (Perrin et al., 2009). In the latter case nickel induced the expression of adhesive-curli-encoding genes (Perrin et al., 2009). In *H. salinarum*, the expression of genes encoding pili-like surface structures was not affected by metal treatment (data not shown). In addition, cations modify the electrical charges of the bacterial surface, resulting in alterations of the bacterial adhesion as shown in *P. fluorescens* exposed to magnesium, zinc or cadmium (McEldowney, 1994; Song and Leff, 2006). The structure of zinc exposed haloarchaeal biofilms did not show any significant differences compared to untreated biofilms. However, biofilms showed an increased survival rate compared to planktonic cells when exposed to zinc, indicating a protective response. The fact that zinc leads to cell death, without changes in cell shape or biofilm structure, underlines the stabilizing effect of zinc on membranes and molecules.

### Heavy Metal Treatment Affects the Transcriptional Activity of Transport Systems

The influence of metal ions on molecular processes in *H. salinarum*, such as metal transporter proteins or general stress proteins (OE3668F and OE4544R) was analyzed by qRT-PCR. The transcription of one of the genes encoding these stress proteins was slightly increased in copper-treated cells, whereas the gene encoding the second stress protein decreased in expression. In contrast, both stress-protein encoding genes were strongly increased in nickel- and slightly increased in zinc-treated cells, suggesting a higher stress level upon nickel- and zinc-treatment. The increased stress level could also explain the growth arrest observed in the presence of 15 mM nickel and 1 mM zinc (MIC), whereas cells are still growing at 5 mM copper (MIC). The strongest decrease was observed in the expression of *pstS*1, encoding a phosphate ABC transporter substrate binding protein with a 14-fold decreased expression in nickel- and an even 127-fold decreased expression in copper-treated cells (Table 3). The strongly decreased transcriptional activity of this transporter gene suggests a metal import function. On the contrary, a strongly enhanced expression was observed in case of the Cu^2+^-exporting ATPase *copA* in copper-, nickel- and zinc-treated cells. The expression of the corresponding copper chaperone OE2044F was also slightly increased. In a microarray analysis of *Halobacterium* sp. strain NRC-1 the expression of the *copA* related *yvgX* was up-regulated in copper and zinc-treated cells (Kaur et al., 2006). Due to a defective growth of the Δ*yvgX* strain only in the presence of copper, the authors concluded *yvgX* to be specific for a copper efflux transport (Kaur et al., 2006). The expression of OE4576F encoding a periplasmic substrate binding protein was strongly increased in copper- and slightly increased in nickel- and zinc-treated cells, suggesting also an efflux function. The expression of OE5245F encoding an ABC transport ATP binding protein, was slightly decreased in copper- and nickel-treated cells, and strongly decreased in zinc-treated cells, suggesting an important role in zinc transport. A divergent behavior in gene expression of the copper-, nickel- and zinc-treated cells was observed for the expression of *znuC* and the ZIP family transporter OE3453R. The expression of *znuC* encoding an ATP-binding protein of the putative zinc uptake system was strongly increased (38 and 18-fold) in nickel- and zinc-treated cells, but decreased in copper-treated cells, indicating that the transporter is not specific to zinc ions. In contrast, the expression of the gene encoding the ZIP family metal transporter, OE3453R, was strongly decreased in copper- and enhanced in nickel- and zinc-treated cells. The term “ZIP family” derives from the first members identified as ‘ZRT, IRT-like Protein,’ including zinc- and iron uptake transporters in *Saccharomyces cerevisiae* and *Arabidopsis thaliana* (Eide et al., 1996; Zhao and Eide, 1996a,b). Members of the ZIP family transport a variety of cations like cadmium, iron, manganese and zinc (Guerinot, 2000). However, the transport of copper and nickel has not yet been reported but might also be possible in case of high metal concentrations.

Divergent behaviors between metal treatment were also shown in the expression of *dppB2* and *dppC2* of the dipeptide permease (Dpp) ABC transport system. The expression decreased in copper- and zinc-treated and increased in nickel-treated cells. The Dpp ABC transporters are involved in the peptides/nickel transport system pathway, implying the increased expression of nickel-treated cells. In addition, they are involved in the bacterial cell communication *via* quorum sensing (QS), responsible for the regulation of gene expression in defense mechanisms, adaptations to changing environments, and biofilm formation. Thus, this transporter might play a crucial role in the observed metal-specific biofilm formation in *H. salinarum*.

## Conclusion

Overall, our analyses identified a variety of metal-specific responses in haloarchaea. In the presence of sub-lethal heavy metal concentrations, cell adhesion was either inhibited or increased. Investigations on cell survival in mature biofilms and in planktonic cells exposed to metals revealed a protective role of biofilms since the amount of dead cells was much lower in biofilms compared to planktonic cells. Microscopic observations of metal-exposed biofilms yielded significant differences in the biofilm architecture, presumably caused by specific resistance mechanisms. In addition, the transcription of genes encoding transport systems showed distinct differences depending on the metals tested.

The metal concentrations tested in our study ranged from 0.005 to 100 mM zinc, 0.5 to 40 mM nickel and 0.5 to 10 mM copper. These concentrations are in the range of the natural exposure of halophilic microorganisms in metal-polluted environments. The natural concentrations vary strongly depending on water level and climate, reaching concentrations of up to 0.7 mM copper, 1 mM nickel, and 11 mM zinc (Kumar et al., 2010; Pereira et al., 2013). Our results show that haloarchaeal biofilms are able to survive in the presence of even higher concentrations of heavy metals. Therefore, biofilms might play an important role in metal immobilization and sequestration processes in metal-polluted environments. Investigations of the resistance-mediating factors in biofilms remain to be important, and might enable the use of biofilms as biotechnological tool in metal remediation (Koechler et al., 2015).

## Author Contributions

SV, SF, and FP planned the study. SV performed the analysis. All authors discussed the results, wrote the manuscript, and approved the final manuscript.

## Conflict of Interest Statement

The authors declare that the research was conducted in the absence of any commercial or financial relationships that could be construed as a potential conflict of interest.
