# Supplementary material for: Heavy Metal Ion Stress on Halobacterium salinarum R1 Planktonic Cells and Biofilms
Source: Front Microbiol. 2018 Dec 18;9:3157. doi: 10.3389/fmicb.2018.03157 (PMC6305349; doi:10.3389/fmicb.2018.03157)
Supplement: Supplementary file 1 [file Data_Sheet_1.PDF]

Heavy Metal Ion Stress on *Halobacterium salinarum* R1 Planktonic Cells and Biofilms

Sabrina Völkel, Sabrina Fröls and Felicitas Pfeifer*

***Correspondence:**
Prof. Dr. Felicitas Pfeifer; pfeifer@bio.tu-darmstadt.de

1 Supplementary Figures

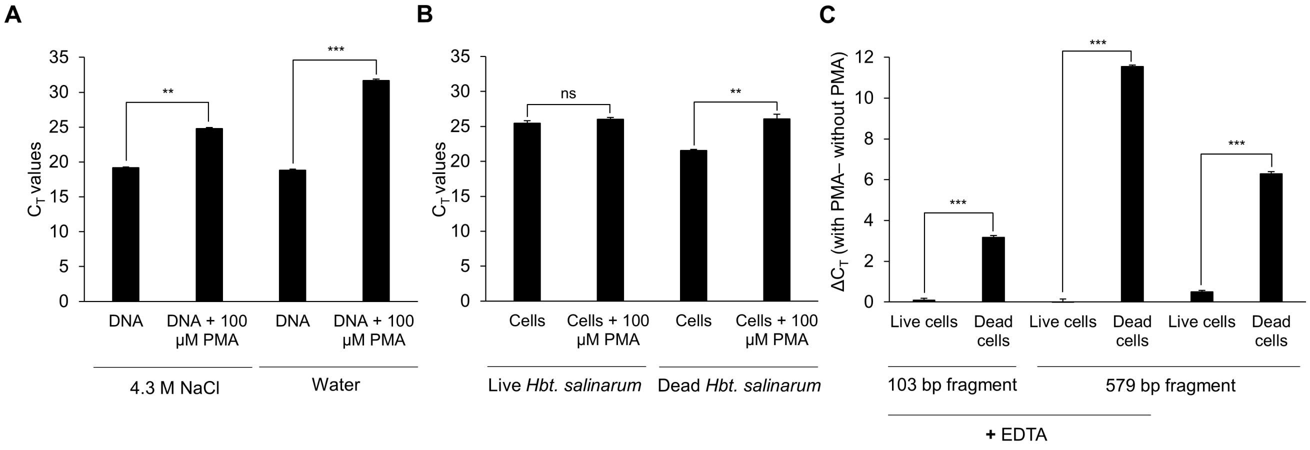

Figure S1. Effect of PMA treatment on the suppression of DNA amplification using qPCR. **(A)** Analysis of PMA treatment with isolated DNA in the presence of 4.3 M NaCl or water (amplification of a 103 bp fragment). **(B)** Effect of PMA treatment on live and dead *Hbt. salinarum* cells in the presence of 4.3 M NaCl (amplification of a 103 bp fragment). **(C)** Effect of PMA treatment on the suppression of DNA amplification of live and dead *Hbt. salinarum* cells by use of EDTA and fragment lengths of 103 bp and 579 bp. Significance of C_T-values was assessed by *t*-test (ns = not significant, * significant = $P < 0.05$, ** highly significant = $P < 0.01$, *** extremely significant = $P < 0.001$).

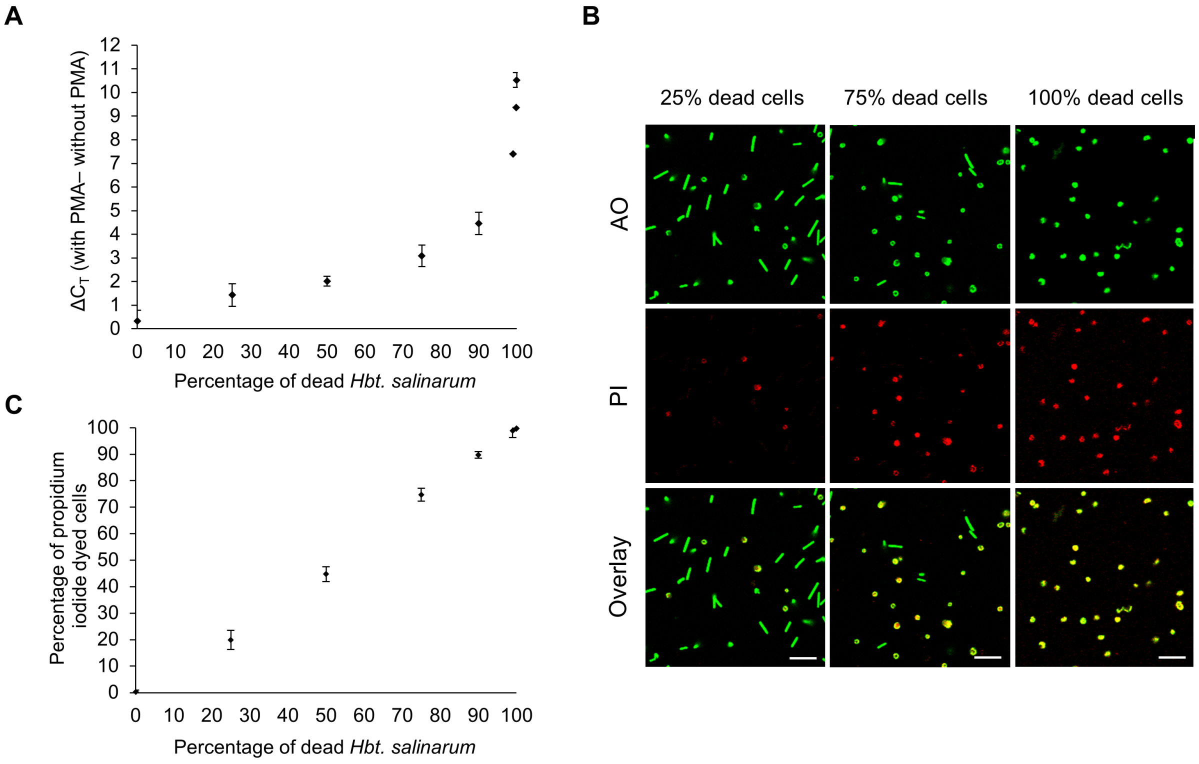

Figure S2. Methods to quantify the amount of dead *Hbt. salinarum* cells. **(A)** Standard of ΔC_T values corresponding to defined percentages of live and dead *Hbt. salinarum* by use of PMA-qPCR. Data based on five independent cultures (N=5). **(B)** Live/dead staining of increasing amounts of dead *Hbt. salinarum* cells (25%, 75%, 100%) using acridine orange (AO) and propidium iodide (PI). Scale bars equal 10 µm in each case. **(C)** Percentage of propidium iodide stained cells after mixing defined ratios of live and dead cells. The fraction of propidium iodide stained cells was examined by fluorescence microscopy and cell counting. For each mixture at least 500 cells were counted.
